# Prognostic model for hepatocellular carcinoma based on anoikis-related genes: immune landscape analysis and prediction of drug sensitivity

**DOI:** 10.3389/fmed.2023.1232814

**Published:** 2023-07-12

**Authors:** Dengyong Zhang, Sihua Liu, Qiong Wu, Yang Ma, Shuo Zhou, Zhong Liu, Wanliang Sun, Zheng Lu

**Affiliations:** ^1^Graduate School, Anhui Medical University, Hefei, China; ^2^Department of General Surgery, The First Affiliated Hospital of Bengbu Medical College, Bengbu, China

**Keywords:** HCC, anoikis, metastasis, immune, prognosis, signature

## Abstract

**Background:**

Hepatocellular carcinoma (HCC) represents a complex ailment characterized by an unfavorable prognosis in advanced stages. The involvement of immune cells in HCC progression is of significant importance. Moreover, metastasis poses a substantial impediment to enhanced prognostication for HCC patients, with anoikis playing an indispensable role in facilitating the distant metastasis of tumor cells. Nevertheless, limited investigations have been conducted regarding the utilization of anoikis factors for predicting HCC prognosis and assessing immune infiltration. This present study aims to identify hepatocellular carcinoma-associated anoikis-related genes (ANRGs), establish a robust prognostic model for HCC, and delineate distinct immune characteristics based on the anoikis signature. Cell migration and cytotoxicity experiments were performed to validate the accuracy of the ANRGs model.

**Methods:**

Consensus clustering based on ANRGs was employed in this investigation to categorize HCC samples obtained from both TCGA and Gene Expression Omnibus (GEO) cohorts. To assess the differentially expressed genes, Cox regression analysis was conducted, and subsequently, prognostic gene signatures were constructed using LASSO-Cox methodology. External validation was performed at the International Cancer Genome Conference. The tumor microenvironment (TME) was characterized utilizing ESTIMATE and CIBERSORT algorithms, while machine learning techniques facilitated the identification of potential target drugs. The wound healing assay and CCK-8 assay were employed to evaluate the migratory capacity and drug sensitivity of HCC cell lines, respectively.

**Results:**

Utilizing the TCGA-LIHC dataset, we devised a nomogram integrating a ten-gene signature with diverse clinicopathological features. Furthermore, the discriminative potential and clinical utility of the ten-gene signature and nomogram were substantiated through ROC analysis and DCA. Subsequently, we devised a prognostic framework leveraging gene expression data from distinct risk cohorts to predict the drug responsiveness of HCC subtypes.

**Conclusion:**

In this study, we have established a promising HCC prognostic ANRGs model, which can serve as a valuable tool for clinicians in selecting targeted therapeutic drugs, thereby improving overall patient survival rates. Additionally, this model has also revealed a strong connection between anoikis and immune cells, providing a potential avenue for elucidating the mechanisms underlying immune cell infiltration regulated by anoikis.

## 1. Introduction

Hepatocellular carcinoma (HCC) accounts for 90% of primary liver malignancies, rendering it the sixth most prevalent neoplasm on a global scale and the fourth leading cause of cancer-related mortality (1, 2). Aflatoxin exposure, hepatitis virus infection, excessive alcohol consumption, type 2 diabetes, and obesity are firmly established as risk factors associated with HCC (3, 4). The distinctive heterogeneity and aggressive behavior exhibited by HCC, coupled with its elevated recurrence rate, contribute to unfavorable prognoses and overall survival (OS) outcomes among patients (5–7). The emergence of metastatic lesions signifies a pivotal event in cancer advancement and continues to pose a substantial hindrance to achieving improved long-term survival (8, 9). While multiple genes have been linked to the metastasis of HCC, their association with the prognosis of liver cancer remains uncertain.

The dissemination of cancer requires the dissociation of cells from the primary neoplasm, their viability during transit, extravasation, and establishment of secondary tumors at remote locations (10). The extracellular matrix (ECM) functions as a scaffold for cellular adhesion, instigates signal transduction, and governs essential cellular processes such as proliferation, migration, differentiation, and viability (11, 12). The acquisition of resistance against anoikis is considered a critical event in initiating and perpetuating metastasis (10, 13), which is also an obligatory prerequisite for both intrahepatic and extrahepatic dissemination of HCC. Upon detachment from the ECM, adherent cells undergo apoptosis, a process referred to as anokis (14). Malignant and highly invasive tumor cells employ diverse mechanisms to surmount anoikis and evade the primary site to establish distant metastases (15–17). While certain crucial functions of apoptosis in tumor advancement and metastasis have been elucidated (18–20), limited research has been conducted to explore the prognostic significance of genes associated with anoikis in HCC. Pseudouridine (Ψ) represents the initial post-transcriptional alteration identified and constitutes a prevalent RNA modification (21). Two distinct modes of pseudouridylation are observed, namely RNA-independent and RNA-dependent. The RNA substrate engages in the formation of complementary base pairs, enabling ncRNA recognition, while catalytic activity is conferred by DKC1 (22, 23). Pseudouridine synthases (PUS) represent a singular enzyme class responsible for catalyzing RNA-independent pseudouridylation, thereby obviating the necessity of RNA template strands (24). Certain instances involve RNA synthases, such as tRNA, exhibiting relatively restricted substrate specificities.

In this investigation, we integrated the GSE14520 and TCGA-LIHC datasets comprising HCC tissues to explore the putative roles of ANRGs. Our aim was to construct an authenticated nomogram capable of prognostic prediction and clinical guidance, accomplished through the development of a scoring, namely “riskScore,” and the categorization of HCC patients according to ANRGs expression patterns. By categorizing patients with HCC based on cellular ANRGs expression, we successfully discerned distinct subgroups that exhibit associations with prognosis and immune infiltration. Employing the LASSO-Cox method, we developed a predictive model for determining the riskScore related to anoikis. Furthermore, through the integration of clinicopathological characteristics, we devised a nomogram for comprehensive risk assessment. Furthermore, we investigated the associations between RNA modifications (Ψ) and the occurrence of anoikis, a programmed cell death process, in relation to the risk of HCC. The predictive accuracy of the nomogram was validated using time-dependent receiver operating characteristic (ROC) and decision curve analysis. Our results indicated a plausible correlation among anoikis, the immune microenvironment, and the prognostic outlook for individuals with HCC.

## 2. Materials and methods

### 2.1. Data and cell lines acquisition

On the TCGA data portal,^1^ which contains 374 LIHC and 50 normal tissue samples, we found gene expression profiles and clinical information for our research, including TNM classification, age, gender, and overall survival. Additionally, we obtained the ICGC dataset from https://icgc.org/, which comprised 240 HCC samples, and the GSE14520 dataset from the GEO database, which contained 221 HCC samples. For analysis, only data that had all available clinical information were used (25, 26). To obtain a comprehensive set of genes associated with anoikis, we use the keywords “anoikis” to search ANRGs in Genecards website. Eventually, a total of 63 ANRGs were retrieved.^2^ HCC cell lines were all obtained from kmcellbank (No. KCB200507YJ; KCB200970YJ).

### 2.2. Consensus clustering with ANRGs

In order to identify distinctive expression patterns associated with regulators of anoikis, we conducted consensus clustering employing the K-means algorithm. The determination of cluster number and stability was accomplished using the “ConsensuClusterPlus” package (27–29). To validate the clustering outcomes, the UMAP algorithm in conjunction with the “ggplot2” R package was employed (30, 31).

### 2.3. GSVA analysis

The Gene Set Variation Analysis (GSVA) analysis was conducted employing the “GSVA” R package (32, 33) using the “c2.cp.kegg.v7.4.symbols.gmt” dataset sourced from the MSigDB database. The determination of statistical significance among subgroups was accomplished through the utilization of the adjusted *p* < 0.05, as provided by the “limma” package (34–36). Subsequent to this, a functional enrichment analysis was performed with the purpose of investigating the functional annotation and enrichment pathways pertaining to differentially expressed genes in hepatocellular carcinoma in relation to ANRGs. T The ClusterProfiler software was employed for the examination of Gene Ontology (GO) and Kyoto Encyclopedia of Genes and Genomes (KEGG) pathways (37). A statistical significance threshold of 0.05 was applied to determine the significance of the results.

### 2.4. LASSO regression analysis

Survival-associated genes were identified through univariate Cox regression analysis. To further refine the selection, LASSO regression analysis was conducted using the “glmnet” package in R, employing cross-validation to determine the optimal penalty regularization parameter (λ) (38). Subsequently, multivariate Cox regression modeling was applied to identify pivotal genes and estimate their corresponding coefficients. The ANRGs risk score was computed f for each patient was calculated using the formula: riskScore = e^ (0.149*FZD7 + 0.395*ADAMTS5 + 0.108*VNN2 + 0.268*MRPL9–0.233*PPARGC1A + 0.111*EPO + 0.127*TSPAN13+ ANP32B*0.561–0.524*TRAC +0.208*RAB328). The predictive performance of the model was assessed through the utilization of Kaplan–Meier curves as well as ROC curves.

### 2.5. Risk score and immune cell infiltration

The composition of infiltrating immune cells was assessed through the utilization of CIBERSORT and ssGSEA (39). The contrasting immune cell types between low-risk and high-risk HCC patients were examined via CIBERSORT.

### 2.6. Chemotherapy response

Protein-drug interactions were investigated through the utilization of Quartata Web (40). To assess the median inhibitory concentration (IC50) values of individual small molecule drugs, the “pRRophetic” R package was employed (41). Briefly, according to the expression levels of 10 ANRGs, HCC patients were divided into high-risk and low-risk groups. Based on the expression patterns of these two groups, drug sensitivity differences between the high-risk and low-risk groups in HCC were evaluated using the drug sensitivity data from “pRRophetic.”

### 2.7. Nomogram

A nomogram was developed employing clinicopathological characteristics. Internal validation encompassed the use of calibration plots to evaluate the precision of the nomogram. In order to assess the predictive efficacy of the nomogram, the Time-C index was employed. Furthermore, DCA method was conducted to ascertain the clinical utility of the intervention (42).

### 2.8. Migration ability test

The ANRGs prognostic model was validated using Huh7 and HepG2 cell lines as experimental models. To evaluate the prognostic accuracy, wound healing assays were performed separately on Huh7 cells exhibiting high-risk scores and HepG2 cells displaying low-risk scores. The migration rates were subsequently compared to determine the relative migration capabilities of these two HCC cell lines.

### 2.9. CCK-8 experiment

In order to validate the accuracy of ANRGs’ drug sensitivity predictions, we selected Erlotinib, which exhibited the most significant differences, for cell viability experiments. HepG2 and Huh7 cells were treated with various concentrations of Erlotinib for 36 h. Subsequently, 10 μL of CCK-8 reagent was added, and the optical density (OD) at 450 nm was measured using a spectrophotometer after a 2-h incubation period, representing the cell viability.

### 2.10. Statistical analysis

R software 4.1.3 was used to conduct the statistical analysis. Graphpad and Image J (version 9.4.0, 1.8.0) were used to analyze the experimental data. T-test was used to assess the difference between the two groups in the cell experiment. *p* < 0.05 were used to determine statistical significance.

## 3. Results

### 3.1. Genetic aberrations in HCC and differential expression of ANRGs

**Figure 1:**
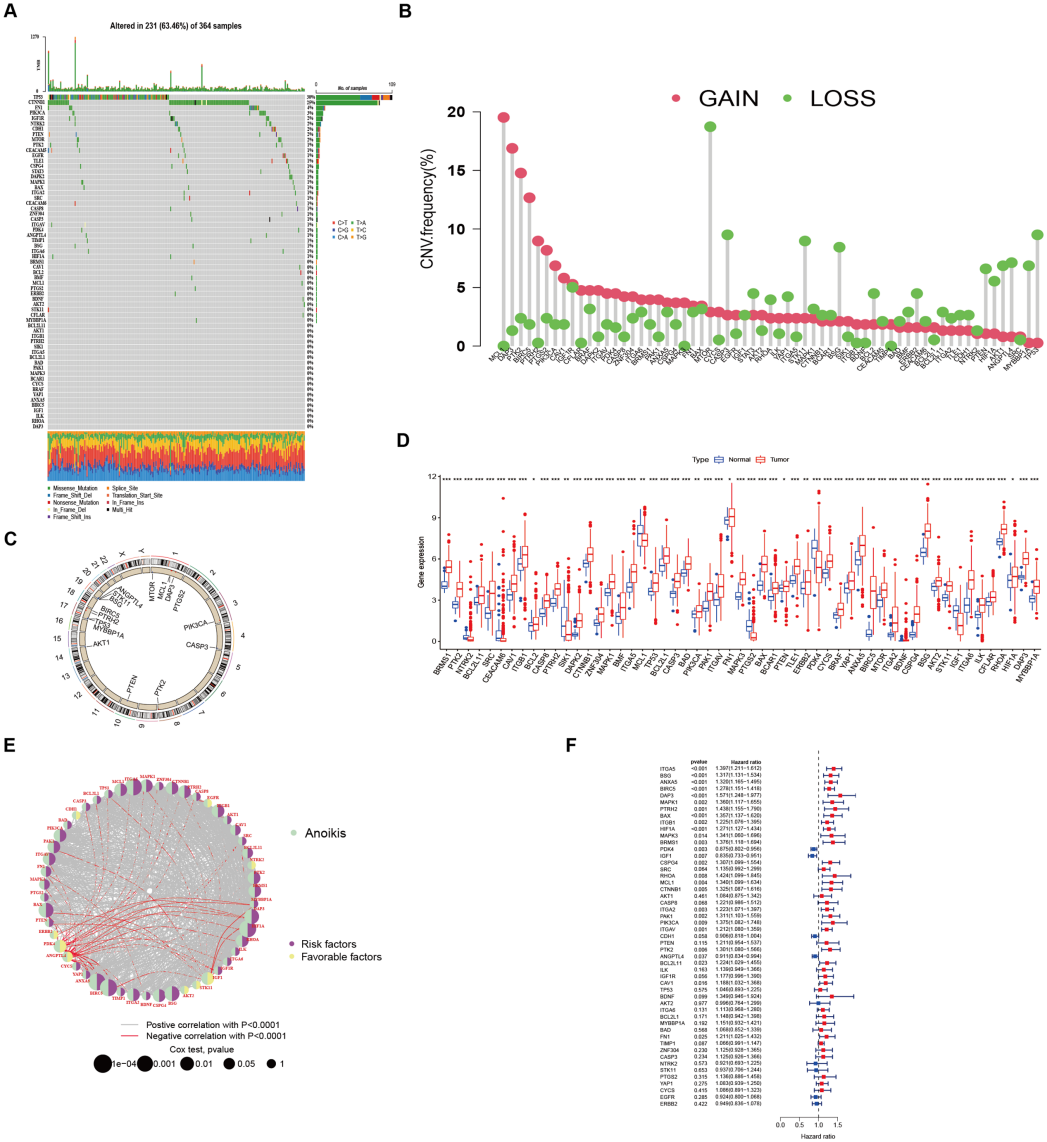
Characteristics and differences of anoikis-related regulators in HCC. **(A)** Mutation profiles of 364 hepatocellular carcinoma samples from the TCGA-LIHC cohort. Each waterfall plot represented mutational information for each anoikis-associated regulator. Corresponding colors were annotated at the bottom to indicate the different mutation types. The top bar graph showed the mutation burden. The numbers on the right side represented mutation frequencies, respectively. **(B,C)** Copy number variations (CNVs) in 16 of the 63 ANRGs in TCGA-LIHC. **(D)** The expressions of anoikis-related regulators between normal tissues (*n* = 50) and HCC tissues (*n* = 364) in TCGA-LIHC cohort (Wilcox test, *p < 0.05; **p < 0.01; ***p < 0.001). **(E)** Network diagram showed the correlations between the 51 regulatory significantly associated with OS. **(F)** Forrest plot of the univariate association of the significantly different genes with OS.

Among the 364 TCGA-LIHC samples analyzed, regulatory mutations associated with anoikis were identified in 231 samples, accounting for approximately 63.46% of the examined samples. Noteworthy, the highest mutation rates were observed in TP53 and CTNNB1 (Figure 1A). Moreover, we detected CNVs in 16 out of the 63 ANRGs within TCGA-LIHC. Predominantly, these alterations manifested as copy number amplifications (Figure 1B), with modifications observed in 16 regulators across different chromosomes (Figure 1C). The expression profiles of the 63 identified regulators were subjected to analysis aimed at discriminating between normal and tumor samples obtained from patients diagnosed with HCC (Figure 1D). Among these regulators, 55 ANRGs exhibited statistically significant alterations. Notably, 49 of them exhibited elevated expression levels in HCC samples. To gain deeper insights into the association between these regulators and patient survival, a new cohort named “LIHC-GSE14520” was generated by integrating clinical data and gene expression data from the GEO and TCGA HCC datasets. Following the data analysis, we proceeded to construct a comprehensive network diagram utilizing the 51 identified regulatory factors (Figures 1E,F).

### 3.2. Identification of HCC patterns through analysis of ANRGs

**Figure 2:**
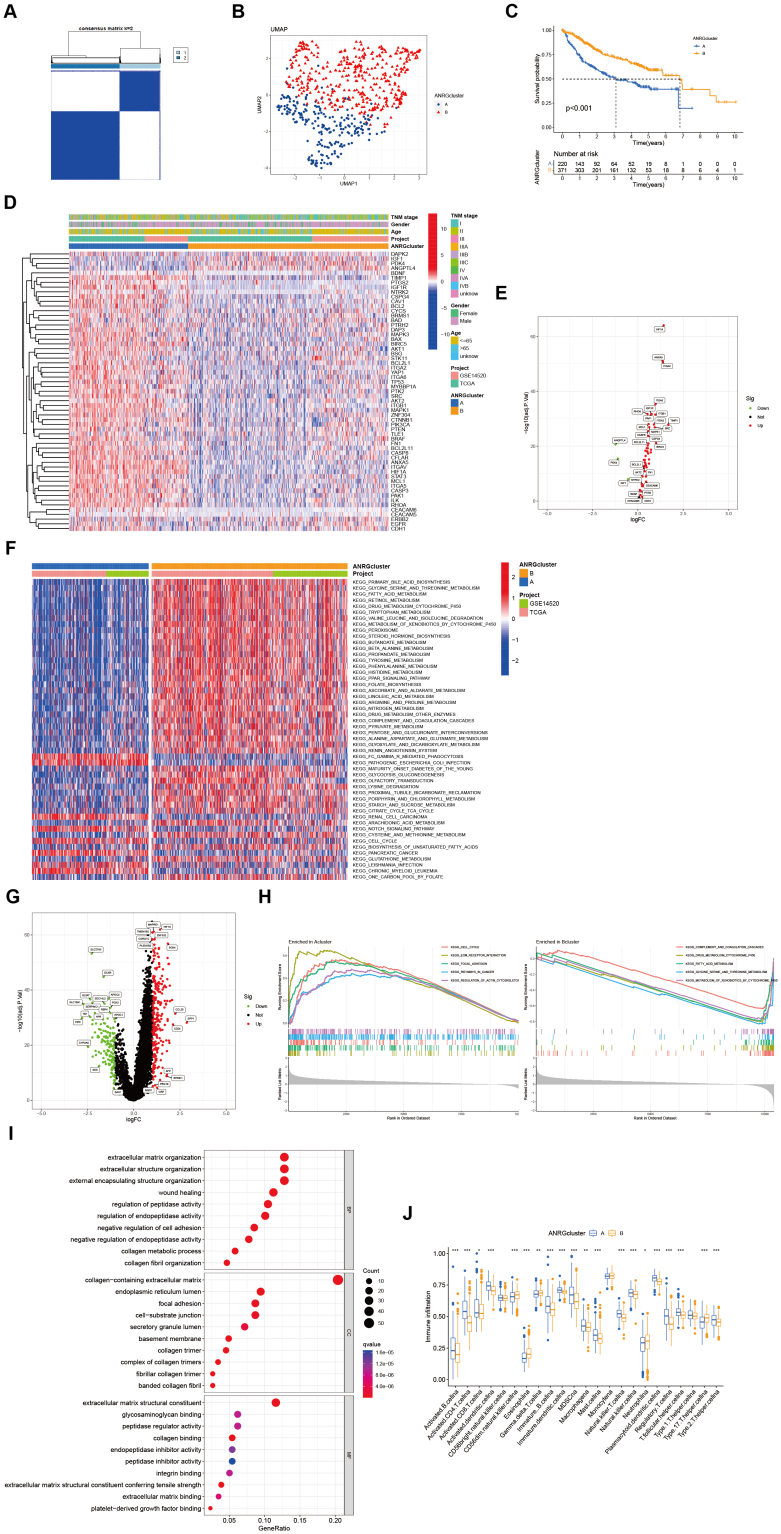
Subgroups of liver cancer related by anoikis-related genes. **(A)** Consensus matrix for *k* = 2 was obtained by applying consensus clustering. **(B)** UMAP distinguished cluster A from cluster B based on the expression of ANRGs. **(C)** Overall survival of cluster A and cluster B (*p* < 0.001). **(D)** Heat map of clinicopathological features of the two subtypes using GSVA enrichment analysis. **(E)** Volcanic diagram of differentially expressed ANRGs. **(F)** Heat map of KEGG pathways of the two subtypes. **(G)** Volcanic diagram of differentially expressed genes (DEG) between cluster A and cluster B. **(H)** GSEA analysis of the most significantly enriched pathways in each of the two clusters, showing the top 5 enriched pathways in cluster A (left) and cluster B (right), individually. **(I)** GO analysis of cluster A vs. cluster B based on 266 upregulated genes obtained from panel **(G)**. **(J)** Box plot showing the abundance of TME infiltrating cells between cluster A and cluster B (Wilcox test, *p < 0.05, **p < 0.01, ***p < 0.001).

Two distinct regulatory patterns were identified through unsupervised clustering using ANRG expression levels. Cluster A comprised 220 cases, while cluster B comprised 371 cases (Figure 2A). UMAP dimensional reduction analysis validated the effective separation of the two clusters based on gene expression levels (Figure 2B). Remarkably, cluster B exhibited a superior survival advantage in comparison to cluster A (Figure 2C). It was also looked at how the cluster related to the clinicopathological traits. When compared to patients in cluster B, patients in cluster A had greater TNM stages (Figure 2D). 53 ANRG genes were found to be substantially different between the two clusters after further gene expression analysis (Figure 2E). The top 50 differential KEGG pathways were shown to be substantially different across the two HCC clusters, according to our GSVA enrichment analysis (Figure 2F). 408 genes were found to be differentially expressed after the DEG analysis of the A-B cluster (Figure 2G). The most significantly enriched pathways were found in both clusters A and B by GSEA enrichment analysis (Figure 2H).

In a total of 266 genes exhibiting upregulation in cluster A were identified in comparison to cluster B, exhibiting a logFC >1 and *p* value <0.05. Subsequently, these genes were subjected to GO enrichment analysis (Figure 2I). The interplay between tumor occurrence and development is substantially influenced by the immune microenvironment. Analysis of the relative abundance of 23 distinct subsets of immune cells within two subpopulations unveils conspicuous infiltration of MDSCs and Tregs in group A, which exhibits diminished rates of survival (Figure 2J).

### 3.3. Development and validation of ANRGs model

**Figure 3:**
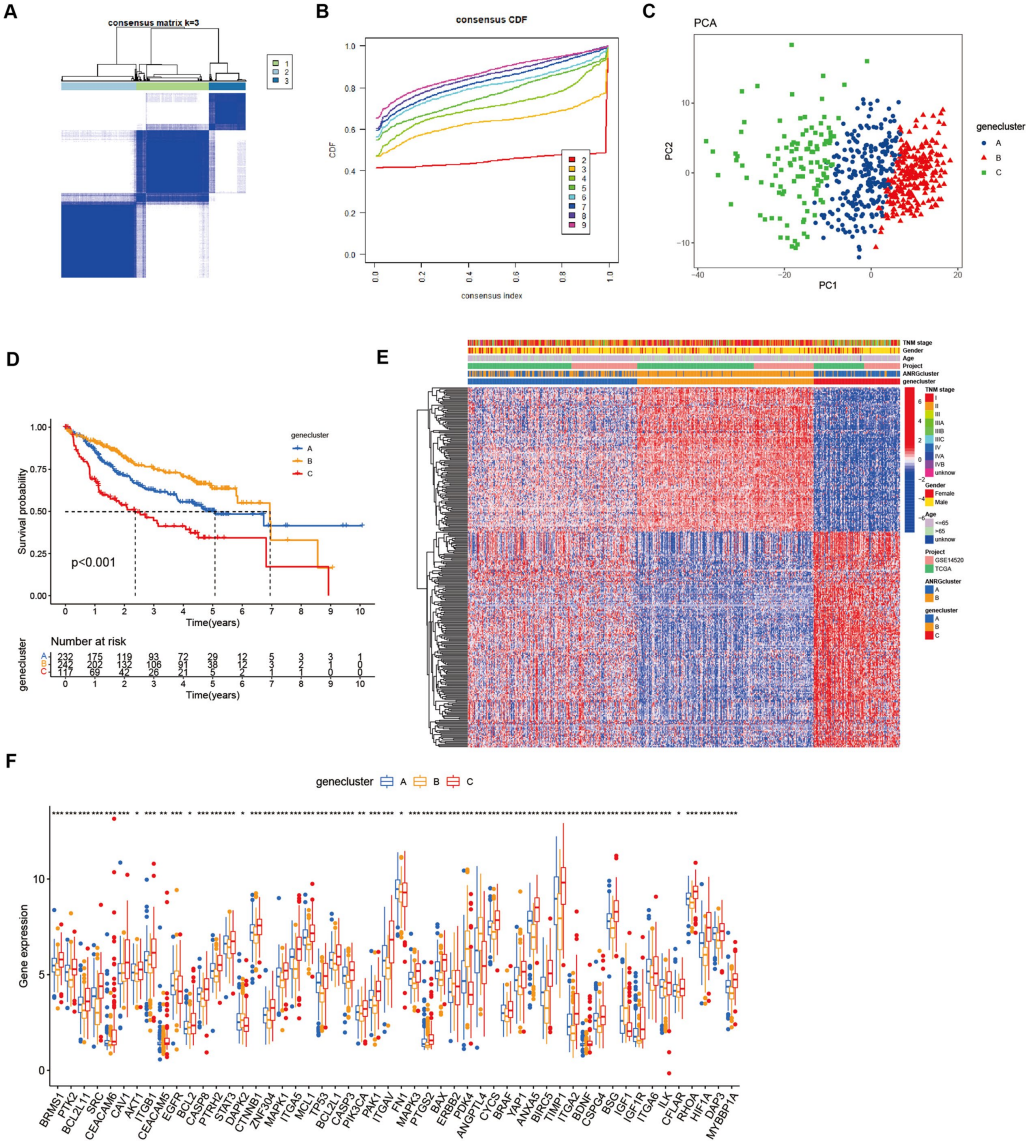
Subgroups of liver cancer related by new gene signature based on two anoikis-related clusters. **(A,B)** Consensus matrix for *k* = 3 was obtained by applying consensus clustering, according to CDF curve. **(C)** Principal component analysis (PCA) for the expression of DEGs to distinguish the three clusters in LIHC-GSE14520 cohort. **(D)** OS in the three clusters in LIHC-GSE14520 cohort. **(E)** Heat map of clinicopathological features of the three subtypes based on DEG. **(F)** ANRGs expression level between cluster A–C (Wilcox test, *p < 0.05, **p < 0.01, ***p < 0.001).

**Figure 4:**
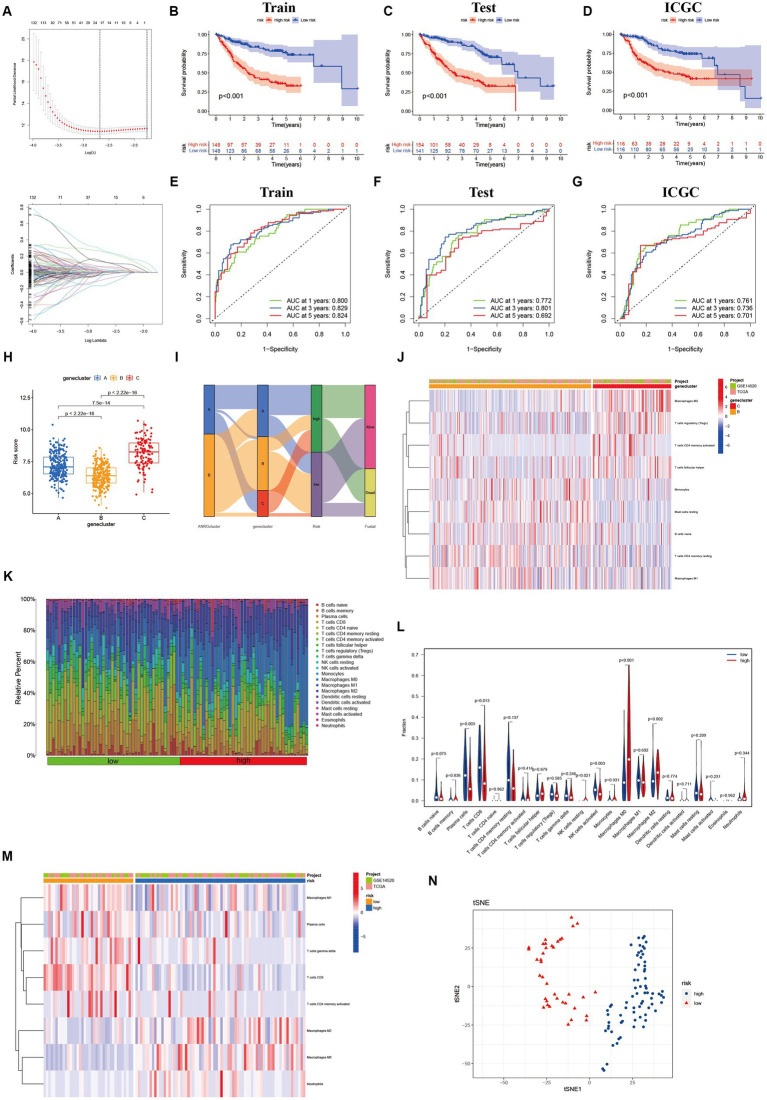
Lasso analysis and Kaplan–Meier curve for the patients in the LIHC-GSE14520 and ICGC cohorts. **(A)** LASSO coefficient profiles of the 312 DEGs based on anoikis-related genes, and the super parameter (*λ*) was obtained based on the minimum standard with 10-fold cross-validation. **(B–D)** Kaplan–Meier plot of high-risk and low-risk in LIHC-GSE14520 cohort and ICGC cohort, which represented training group **(B)**, test group **(C)** from LIHC-GSE14520 cohort and ICGC validation group **(D)**, individually. **(E–G)** AUC time-dependent ROC curves for OS in training, test and validation cohort. **(H)** The level of “riskScore” in different clusters. **(I)** The Sankey diagram of the relationship between different clusters and living risk. **(J)** Immune infiltration between cluster B and cluster C. **(K–M)** Immune cell infiltration in different risk groups based on DEGs in the two ANRGs clusters. **(N)** The tSNE analysis demonstrated the differentiation between high and low risk groups.

To streamline the clinical management of hepatocellular carcinoma (HCC), our objective was to construct a subtype-specific scoring system based on patient characteristics. To accomplish this, we performed univariate Cox regression analysis to identify a set of 312 genes associated with survival. Subsequently, we subjected these prognosis-related genes to consensus clustering analysis, resulting in the discovery of three distinct regulatory patterns (Figures 3A,B). Notably, principal component analysis (PCA) unveiled substantial expression discrepancies of the aforementioned genes among these three HCC subtypes (Figure 3C). Moreover, each subtype exhibited unique overall survival outcomes (Figure 3D), affirming the prognostic reliability of the identified genes. Additionally, a comparative examination of clinical parameters between clusters A and C revealed significant differences (Figure 3E). Importantly, among the 59 genes analyzed, a striking 53 ANRGs displayed significant variations across the three regulatory patterns (Figure 3F). To establish a measurable framework applicable to individual patients, a LASSO-Cox regression analysis was subsequently executed on the set of differentially expressed genes, leveraging the training cohort. Consequently, a total of 10 risk-associated genes were identified (Figure 4A). Utilizing these 10 genes, a risk scoring system was devised for each HCC sample. Through Kaplan–Meier analysis, a noteworthy survival advantage was observed in the low-risk group compared to the high-risk group, as validated in both the training and test cohorts (Figures 4B,C). Furthermore, the derived risk score exhibited significant predictive value for 1-year, 3-year, and 5-year survival rates in HCC patients (Figures 4E,F). Ultimately, the ANRGs model was subjected to validation using an independent ICGC cohort (Figures 4D,G).

### 3.4. Immune infiltration

**Figure 5:**
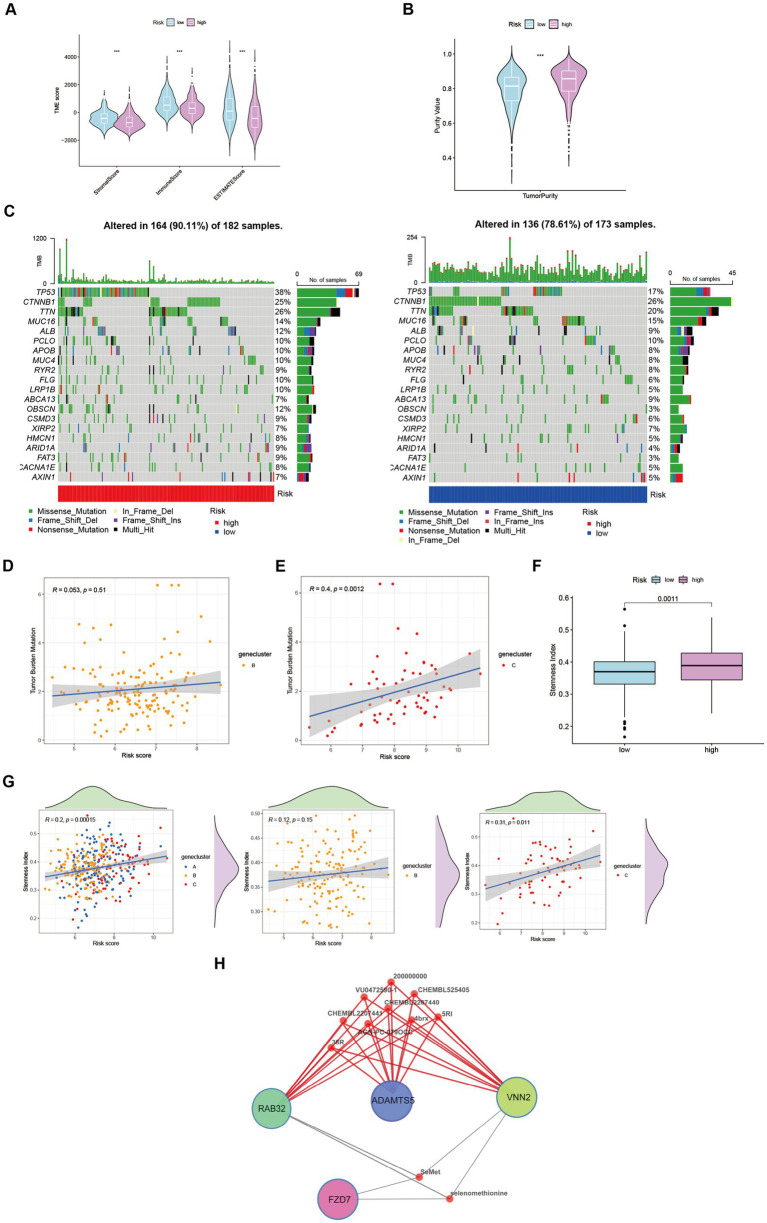
Characteristics and differences between different risk-groups in HCC. **(A)** The score of components in tumor microenvironment (Wilcox test, ***p < 0.001). **(B)** Influence of tumor purity to risk score (Wilcox test, ***p < 0.001). **(C–E)** Mutation profiles of TCGA-LIHC cohort with different risk statue as well as cluster B and cluster C (Spearman test, *p* = 0.51; *p* = 0.0012). **(F)** Stemness Index in different risk groups (Wilcox test, *p* = 0.0011). **(G)** The relationship between stemness index and risk score in LIHC-GSE14520 cohort (Spearman test). **(H)** Potential targeting drugs prediction via QuartataWeb Server.

Through its function in immune evasion, TME, and particularly the immune system, is crucial to the development of malignancies. Notably, there were notable variations between clusters B and C in the riskScores of three clusters (Figures 4H,I). We next looked into the immunological infiltration between these groups, and the results revealed that cluster C had the highest levels of Tregs and macrophages M0 (Figure 4J). Additionally, we determined each patient’s relative number of various immune cells and rated HCC patients according to their riskScore values (Figure 4K). As expected, the high-risk group had much greater levels of macrophages M0 and M2 (Figure 4L). To explore variations in immune cell populations among different risk groups, we applied a screening criterion given the continuous nature of riskScore in our cohort. This screening approach enabled us to identify noteworthy changes specifically within macrophages and CD8 T cells (Figure 4M). Furthermore, employing t-SNE analysis on the dataset comprising differential expression profiles of immune cells, we observed a discernible segregation of samples into three distinct subgroups (Figure 4N). Furthermore, Moreover, the TME encompasses the extracellular matrix (ECM), a critical constituent that substantially influences the migratory capability, adhesion propensity, and angiogenic processes of neoplastic cells. In order to ascertain the tumor purity for individual specimens, we employed the “Estimate” R package to evaluate the stromal and immune cell scores. Remarkably, HCC patients classified as high-risk exhibited diminished immune cell quantities and elevated tumor purity levels compared to their low-risk counterparts (Figures 5A,B).

Tumor mutational burden (TMB) serves as a biomarker for immunotherapy in diverse solid malignancies. We performed an analysis of TMB variations within distinct risk groups and clusters (Figure 5C). TMB exhibits predictive value for immune checkpoint inhibitor (ICI) efficacy, suggesting heightened responsiveness of high-risk patients to ICI treatment. Additionally, cluster C may potentially derive greater benefits from ICI therapy compared to cluster B (Figures 5D,E). Moreover, cancer stem cells (CSCs) possess the capacity for self-renewal, differentiation, and contribute to HCC development and treatment resistance. A significant upregulation of mRNA-based stemness index (SI) was observed in the high-risk group (Figure 5F). Furthermore, a positive association between risk score and SI was noted, with cluster C displaying a more pronounced correlation relative to cluster B (Figure 5G). To identify potential therapeutic agents for the high-risk group, QuartataWeb Server was employed, considering the unfavorable prognosis linked to this subgroup (Figure 5H).

### 3.5. Prediction of drug response

**Figure 6:**
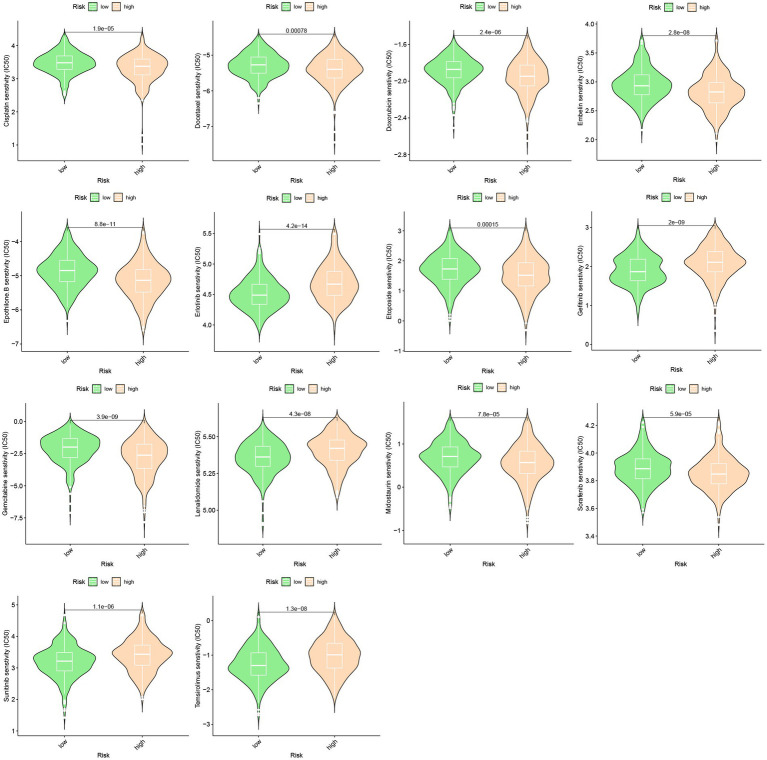
Drug sensitivity between high-risk group and low-risk group.

A predictive model was developed to estimate the response of HCC patients to chemotherapeutic treatment, leveraging the notable variances in gene expression profiles observed across distinct risk categories. The pRRophetic software was used in our method to foresee variations in the susceptibility of tumors to anticancer drugs using gene expression data collected from various risk categories. The results of our investigation revealed a significantly elevated probability of response to axitinib in the high-risk group, whereas the low-risk group exhibited a greater propensity for responding to epothilone B (Figure 6).

### 3.6. Association between ANRGs and Pseudouridine (Ψ)

**Figure 7:**
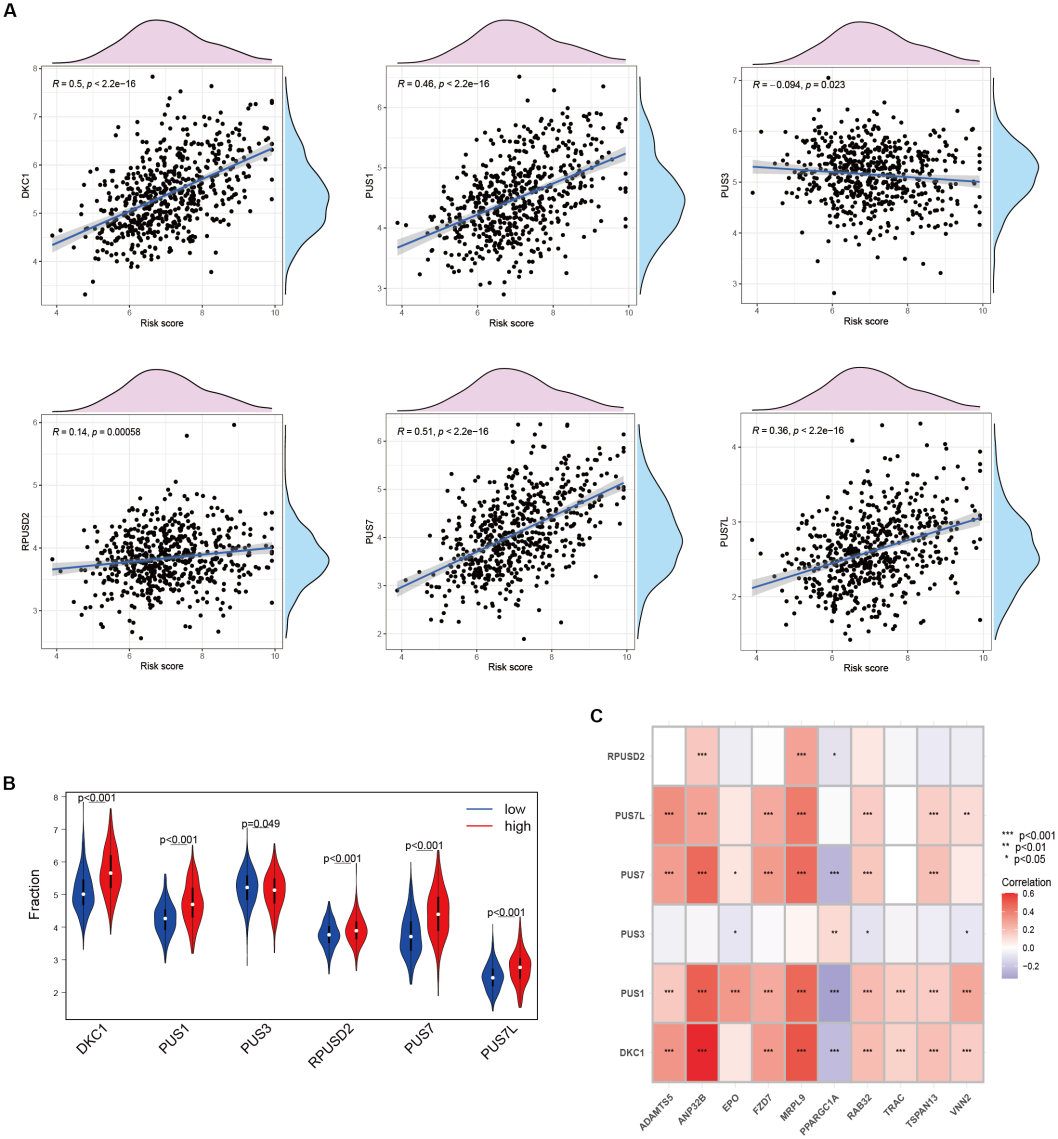
Relationship between Anoikis and Pseudouridine (Ψ) in HCC. **(A)** The relationship between Ψ regulator and risk score in LIHC-GSE14520 cohort. **(B)** The expressions of Ψ regulators with different risk status (Wilcox test). **(C)** Correlation of ANRGs and Ψ regulators (Wilcox test, *p < 0.05; **p < 0.01; ***p < 0.001).

Upregulation of RNA modifiers is linked to the prognosis of tumor illnesses. rRNA, snoRNA, and snRNA are all modified during the RNA pseudouridylation process. Through the investigation of the correlation between risk scores and the expression patterns of pseudouridylation genes (Figure 7A), as well as the comparative analysis of gene expression levels between high-risk and low-risk cohorts (Figure 7B), we have elucidated the possible involvement of in anoikis resistance in HCC patients. Our results reveal a robust association between genes involved in pseudouridylation and aberrantly expressed ANRGs, thereby implying a notable involvement of pseudouridylation in the prognostic implications for HCC patients’ survival outcomes (Figure 7C).

### 3.7. Establishing the HCC nomogram

**Figure 8:**
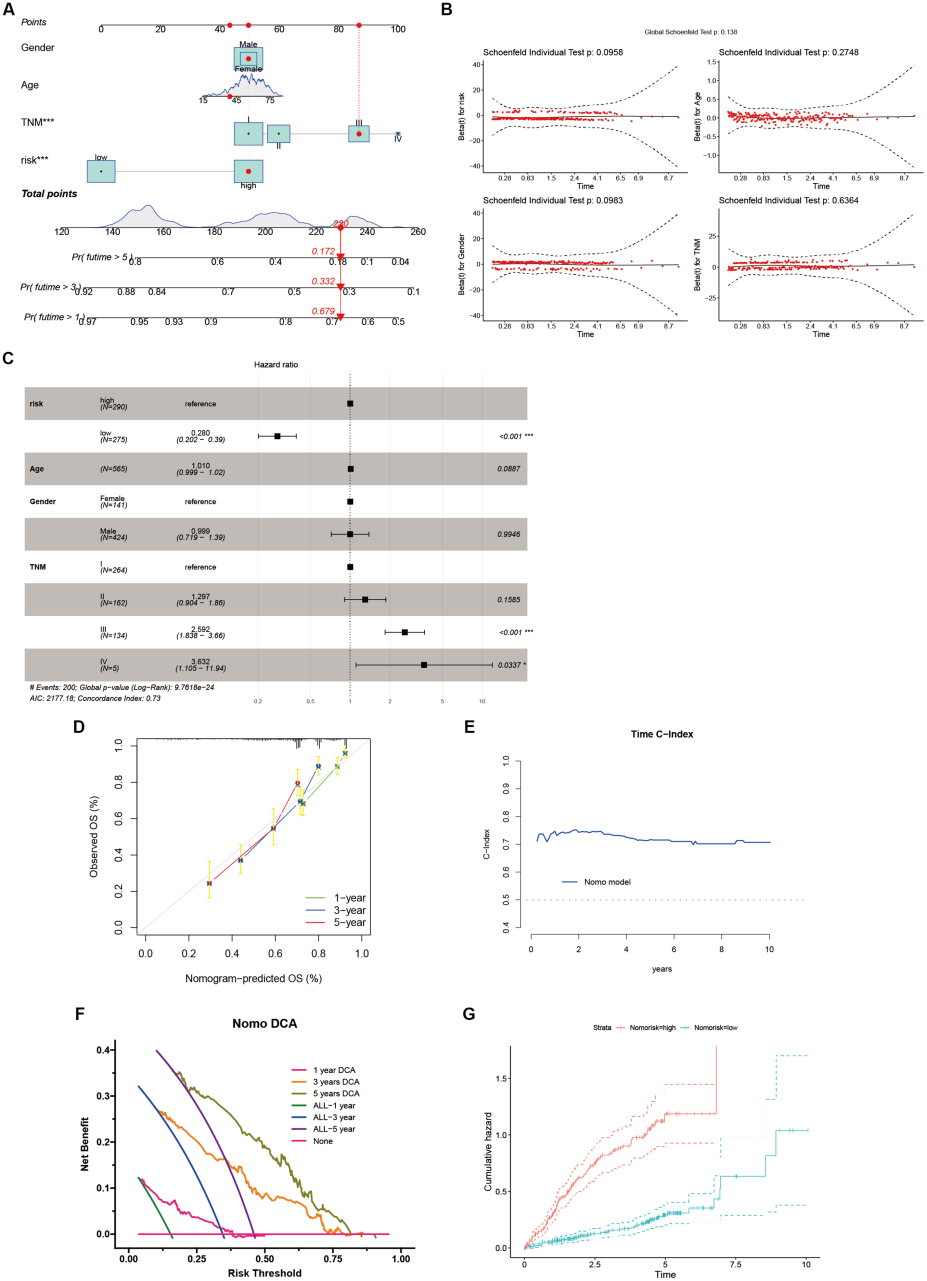
Identification and Verification of Nomograms. **(A)** A nomogram prediction ability at 1, 3, and 5 years. **(B,D,E)** Schoenfeld Residuals Test was performed to verify the validity of nomogram, and calibration plot for internal validation of the nomogram. Time C-index evaluated the predictive performance of nomogram at different times. **(C)** Forest plot summary of multivariable Cox regression analyses of the clinical features as well as risk score in the LIHC-GSE14520 cohort. **(F)** DCA curves of the nomogram for 1-, 3- and 5- year OS in LIHC-GSE14520 cohort indicated the clinical decision-making benefits of this model. **(G)** Cumulative hazard curve represented the probability of survival over time progression.

We devised a nomogram to forecast the overall survival rate with the aim of exploring the therapeutic applications of riskScore in determining the prognosis of individuals afflicted with HCC (Figure 8A). The nomogram encompasses four autonomous prognostic factors, wherein riskScore and TNM classification play principal roles (Figure 8C). Verification of the model’s accuracy and the nomogram’s reliability was achieved through the application of the Schoenfeld Residuals Test and calibration curve analysis (Figures 8B,D). The predictive capacity of the integrated nomogram was found to be significantly superior, as evidenced by the time-dependent ROC curve analysis. The time-C index surpassed 0.7, affirming its robustness in forecasting (Figure 8E). Furthermore, decision curve analysis (DCA) corroborated that the nomogram represents the most precise approach to prognosticate HCC patients’ survival (Figure 8F). The cumulative hazard curve demonstrated a gradual escalation in the jeopardy of overall survival among patients exhibiting elevated scores in the nomogram (Figure 8G), thereby underscoring the significance of employing the nomogram in conjunction with risk scores derived from ANRGs as a potent approach to prognosticate patient outcomes in the realm of clinical application.

### 3.8. Migration ability between HCC cell lines in different risk scores

**Figure 9:**
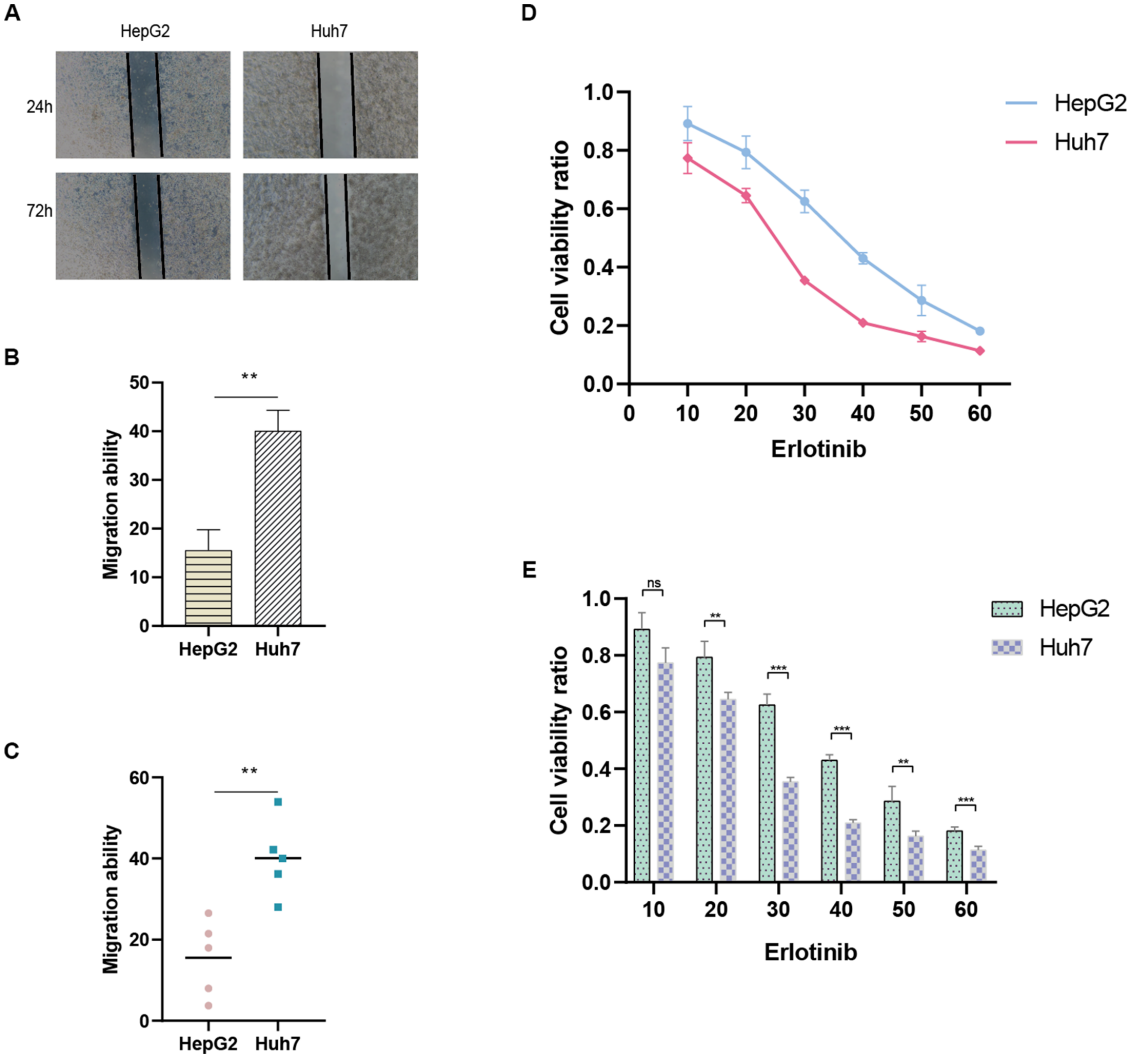
Wound healing assay. **(A)** Migration ability detected via wound healing assay. **(B,C)** Results of migration ability between HepG2 and Huh7 cell lines. **(D,E)** Drug sensitivity to Erlotinib in HCC cell lines.

The predictive performance of the ANRGs prognostic model was assessed using Huh7 and HepG2 cell lines. To evaluate their migratory abilities, wound healing experiments were conducted on Huh7 cells exhibiting high-risk scores and HepG2 cells exhibiting low-risk scores. A comparative analysis of the migration rates between these two HCC cell lines was conducted. Our findings demonstrated a significantly diminished migration rate in HepG2 cells with low-risk scores in contrast to Huh7 cells with high-risk scores (*p* < 0.01; Figures 9A–C). These results suggest that a high-risk score is indicative of heightened metastatic potential in tumors, which may contribute, in part, to the unfavorable prognosis observed in HCC patients. These findings are consistent with the outcomes derived from our ANRGs prognostic model.

### 3.9. Validation of drug sensitivity in HCC cell lines

To evaluate drug sensitivity, the CCK-8 assay was employed. Higher absorbance values indicate stronger cellular viability, thus reflecting decreased sensitivity to the tested drug. We observed differential sensitivity of HepG2 and Huh7 cells to Erlotinib at various concentrations (Figures 9D,E). Huh7 cells with higher risk scores exhibited greater sensitivity to Erlotinib, as evidenced by lower OD values, compared to HepG2 cells with lower risk scores. These findings align with our drug prediction outcomes, thereby affirming the reliability of the predictive model.

## 4. Discussion

Globally, HCC continues to be a particularly deadly kind of cancer (43). Given the intricate molecular pathways implicated, enhancing the prognosis of HCC patients through singular targeted pathways or drug interventions proves arduous. The major contributors to diminished survival rates among these patients are metastasis and postoperative recurrence. Although several genetic markers with predictive potential for HCC have been discovered (44–46), their count remains inadequate. Hence, there exists a pressing requirement to identify supplementary biomarkers exhibiting robust predictive efficacy to expand the pool of potential candidates.

Anoikis is a form of regulated cellular apoptosis triggered by the detachment of cells from the appropriate extracellular matrix, thereby disrupting integrin-mediated adhesion. This process serves as a vital mechanism in safeguarding tissue homeostasis and development by inhibiting the growth and attachment of dysplastic cells to unsuitable substrates (47). Dysregulation of anoikis, characterized by resistance to anchorage-dependent growth and epithelial-mesenchymal transition, has garnered considerable scientific interest owing to its implication in tumor progression and the metastatic dissemination of malignant cells. In HCC, multiple signaling cascades possess the capability to disrupt the phenomenon of anoikis resistance, consequently leading to the attenuation of tumor metastasis. Consequently, therapeutic intervention strategies targeting genes associated with anoikis have surfaced as a promising avenue for surmounting the advancement and metastatic potential of HCC. Employing a polygenic profiling approach, encompassing multiple genes, affords a comprehensive assessment of the intricate interplay among diverse factors governing tumor pathology and the acquisition of anoikis resistance. This innovative approach holds substantial promise in providing crucial insights into tumor biology, thereby furnishing indispensable support for clinical decision-making in the epoch of precision medicine within the domain of oncology (48).

In this investigation, the expression patterns of genes associated with HCC in relation to anoikis were initially ascertained (Figure 1D). Subsequently, a comprehensive screening was conducted to identify ANRGs that exhibit associations with the prognosis of HCC (Figures 1E,F). By employing KEGG and ssGSEA for further analysis, differential enrichment of pathways was discovered, indicating a potential influence of these anoikis-associated genes on the survival outcomes of HCC patients by modulating these pathways (Figures 2C,F,H). Leveraging the Lasso technique, we successfully identified ten ANRGs as crucial genes for the prognostic model, enabling the categorization of HCC patients into high-risk and low-risk groups (Figure 4A). Immunotherapy and the advancement of cancer treatment rely heavily on the immune system (49). Moreover, the modulation of cytokine equilibrium significantly influences the progression of the disease (50). Ongoing investigations are currently focused on exploring the potential of exosome-based immunotherapy (51). In addition, the development of deep learning models is underway to predict the efficacy of immunotherapy (52). Within the TME, a diverse repertoire of chemokines and cytokines is generated by both immune and cancer cells, playing pivotal roles in regulating tumor progression and expansion. By utilizing the Cibersort software, we conducted an additional investigation into the immune infiltration patterns within the high-risk and low-risk cohorts (Figures 4K–M). This analysis revealed that the immunosuppressive phenotype characterizes this particular subgroup.We have identified a cohort of ten genes displaying robust associations with the risk of cancer (Figures 4B–D).

In the course of our investigation, a collection of eight genes displaying strong associations with cancer risk has been identified. Previous studies have established multiple connections between these genes and the growth and advancement of tumors. Notably, Arechederra M and co-authors have provided evidence showcasing the pivotal role of ADAMTSL5, a protein synthesized by liver cancer cells, in the formation of tumors. Targeting this gene effectively has demonstrated reductions in both *in vitro* and *in vivo* tumor growth (53). Furthermore, in the context of colorectal cancer, heightened expression of ADAMTS5 has emerged as a significant indicator of lymphatic infiltration and metastasis (54). According to research conducted on HCC cell lines, FZD7, which is overexpressed in gastric, esophageal, and HCC (55), directly interacts with Wnt signaling to activate the traditional Wnt/−linked protein pathway (56). Epithelial mesenchymal transition (EMT), which is triggered by this activation, encourages HCC to migrate and invade more widely (57). The FZD7/Wnt axis may be blocked to drastically reduce the production of tumor-related proteins and to slow the HCC development (58). Furthermore, FZD7 exhibits anti-apoptotic actions in HCC (59). Despite having received less attention in oncology research, MRPL9 has been discovered to have an oncogenic characteristic in breast cancer (60). The VNN2 protein is essential for cell transendothelial migration and is linked to non-adhesive proliferation, which raises the possibility that it contributes to tumor anoikis resistance (61). VNN2 was discovered to be up-regulated in a human metastasizing esophageal cancer cell line (T.Tn-AT1) in comparison to the parental non-metastasizing cell line (T.Tn), emphasizing its significance in metastasis (62). Rab32, on the other hand, is connected to mTORC1 signaling and the stimulation of -catenin/TCF signaling and expressed in a variety of secretory epithelial cells (63). The proliferation, migration, and metastasis of esophageal squamous cell carcinoma (ESCC) cells have been demonstrated to be inhibited by suppression of RAB23 expression but promoted by overexpression of RAB23 (64). Tetraubiquitin superfamily member TSPAN13, commonly known as NET-6, has been linked to a number of biological activities, including motility and metastasis (65). TSPAN13 expression has been demonstrated to be suppressed by certain miRNAs (66–68), which results in mesenchymal–epithelial transition (MET) and less tumor invasion and growth (69) The pleiotropic growth factor erythropoietin (EPO), on the other hand, has been shown to encourage the development of soft agar colonies in human hepatoma cells, indicating that it may play a part in conferring anoikis-resistance (70). Contrarily, PPARGC1A expression has been found to be downregulated in HCC, and *in vitro* and *in vivo* tests have demonstrated that upregulation of PPARGC1A can successfully prevent HCC cell invasion and migration by blocking the Wnt/−catenin/PDK1 axis and thereby inhibiting aerobic glycolysis (71).

Pseudouridine (Ψ) stands out as a prominent RNA modification and represents the inaugural post-transcriptional alteration to have been identified. Unlike methylation, Ψ exhibits irreversibility within mammalian systems (72). DKC1 assumes an indispensable role as a constituent of the telomerase complex, with its participation being crucial in the post-transcriptional processing of precursor rRNA, thereby exerting a pivotal influence on the progression of tumor cells (73). During our investigation, we assessed the expression levels of DKC1, PUS1, and PUS7 among patients diagnosed with HCC, revealing a robust and significant correlation between pseudouridine and anoikis (Figure 7A). Significantly elevated expression of these genes was observed in the high-risk cohort (Figure 7B), implying a vital contribution of Ψ in the advancement of HCC and its metastatic dissemination. Furthermore, our correlation analysis has provided preliminary evidence suggesting a plausible association between Ψ and anoikis in the context of HCC (Figure 7C).

Sample classification using established gene expression characteristics is a widely employed methodology (74, 75). In our study, we utilized a comparable strategy to classify HCC patients by analyzing the expression profiles of cellular regulators associated with anoikis, in conjunction with clinicopathological indicators (Figure 8A). Our results revealed substantial differential expression of these regulators among distinct subgroups, and their expression patterns were associated with diverse prognoses, substantiating the effectiveness of our ten-gene signature in discerning patient outcomes. This signature holds the potential to aid clinicians in formulating personalized therapeutic approaches. Furthermore, the decision curve analysis indicates that the nomogram constructed using the ten-gene signature could yield long-term advantages for hepatocellular carcinoma patients (Figure 8F).

Finally, to validate the reliability of the ANRGs model, we conducted wound healing assay and CCK-8 assay to examine the migration ability and drug sensitivity of different risk-scored HCC cell lines, HepG2 and Huh7. We observed that the migration ability of the high-risk Huh7 cells was significantly higher than that of the low-risk-scored HepG2 cells (Figures 9A–C), which partly explains the poor prognosis of high-risk HCC patients. Furthermore, Huh7 cells exhibited a significantly higher response to Erlotinib compared to HepG2 cells (Figures 9D,E), confirming the accuracy of our drug prediction (Figure 6).

Although our riskScore model and the corresponding nomogram demonstrate enhanced predictive efficacy, the cellular heterogeneity implies that investigating the influence of ANRGs on hepatocellular carcinoma advancement and prognosis at the individual cell level could provide heightened precision. Furthermore, the utilization of a restricted dataset in this investigation necessitates the inclusion of a more extensive sample size to adequately calibrate the predictive model.

## 5. Conclusion

Our investigation has devised a signature composed of ten genes and accompanying nomograms, which hold potential utility for clinicians in the individualized selection of chemotherapy regimens for patients with HCC. The ten-gene signature, intricately linked to anoikis, exhibits remarkable efficacy in prognosticating the survival outcomes of HCC patients. Additionally, the nomogram derived from this predictive model holds promise as a valuable tool for healthcare professionals in formulating personalized treatment strategies within clinical contexts. Future explorations into the molecular underpinnings of resistance to anoikis hold significant clinical implications, with the potential to provide a novel precision medicine approach for HCC.

## Data availability statement

The datasets utilized in the present investigation can be accessed from the TCGA database (http://cancergenome.nih.gov/), GEO (https://www.ncbi.nlm.nih.gov/geo/), and the ICGC (https://icgc.org/). All raw data are available at https://www.jianguoyun.com/p/DexQH4oQovD_ChjWlvwEIAA.

## Author contributions

ZLu, WS, and DZ conceived the study. DZ drafted the manuscript. DZ, SL, QW, and YM analyzed and visualized the data. SZ, ZLiu, and WS performed the literature search and collected the data. ZLu and WS helped with the final revision of this manuscript. All authors reviewed and approved the final manuscript.

## Funding

This study was supported by Anhui University Natural Science Research Project (KJ2021A0731), the sixth batch of “special support plan” leading talent projects in Anhui Province, the Natural Science Foundation of Bengbu Medical College (2020byzd106), and the Overseas visiting and training program for outstanding colleges teachers in Anhui (gxgwfx2022024).

## Conflict of interest

The authors declare that the research was conducted in the absence of any commercial or financial relationships that could be construed as a potential conflict of interest.

## Publisher’s note

All claims expressed in this article are solely those of the authors and do not necessarily represent those of their affiliated organizations, or those of the publisher, the editors and the reviewers. Any product that may be evaluated in this article, or claim that may be made by its manufacturer, is not guaranteed or endorsed by the publisher.

## Footnotes

^1^ https://portal.gdc.cancer.gov/

^2^ https://www.genecards.org/
